# Optical Screening and Classification of Drug Binding to Proteins in Human Blood Serum

**DOI:** 10.1021/acs.analchem.3c03713

**Published:** 2023-11-08

**Authors:** Samantha H. Rutherford, Christopher D. M. Hutchison, Gregory M. Greetham, Anthony W. Parker, Alison Nordon, Matthew J. Baker, Neil T. Hunt

**Affiliations:** † WestCHEM, Department of Pure and Applied Chemistry, University of Strathclyde, Technology and Innovation Centre, 99 George Street, Glasgow G1 1RD, U.K.; ‡ STFC Central Laser Facility, Research Complex at Harwell, Rutherford Appleton Laboratory, Harwell Campus, Didcot OX11 0QX, U.K.; § WestCHEM, Department of Pure and Applied Chemistry and CPACT, University of Strathclyde, 295 Cathedral Street, Glasgow G1 1XL, U.K.; ∥ School of Medicine and Dentistry, University of Central Lancashire, Fylde Rd, Preston PR1 2HE, U.K.; ⊥ Department of Chemistry and York Biomedical Research Institute, University of York, Heslington, York YO10 5DD, U.K.

## Abstract

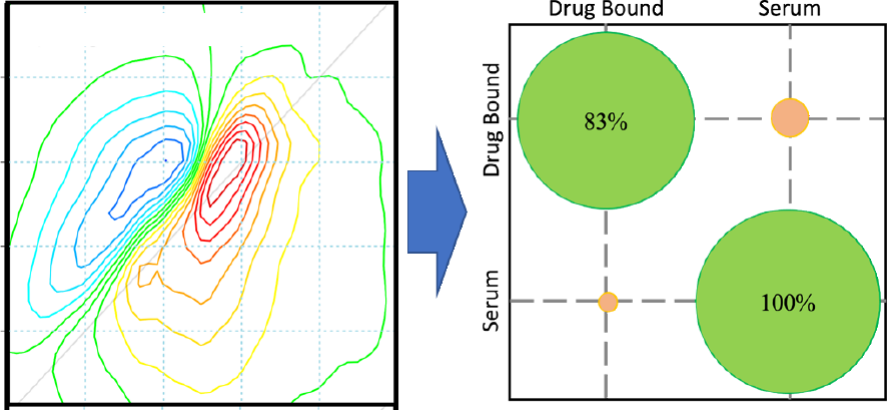

Protein–drug interactions in the human bloodstream are important factors in applications ranging from drug design, where protein binding influences efficacy and dose delivery, to biomedical diagnostics, where rapid, quantitative measurements could guide optimized treatment regimes. Current measurement approaches use multistep assays, which probe the protein-bound drug fraction indirectly and do not provide fundamental structural or dynamic information about the *in vivo* protein–drug interaction. We demonstrate that ultrafast 2D-IR spectroscopy can overcome these issues by providing a direct, label-free optical measurement of protein–drug binding in blood serum samples. Four commonly prescribed drugs, known to bind to human serum albumin (HSA), were added to pooled human serum at physiologically relevant concentrations. In each case, spectral changes to the amide I band of the serum sample were observed, consistent with binding to HSA, but were distinct for each of the four drugs. A machine-learning-based classification of the serum samples achieved a total cross-validation prediction accuracy of 92% when differentiating serum-only samples from those with a drug present. Identification on a per-drug basis achieved correct drug identification in 75% of cases. These unique spectroscopic signatures of the drug–protein interaction thus enable the detection and differentiation of drug containing samples and give structural insight into the binding process as well as quantitative information on protein–drug binding. Using currently available instrumentation, the 2D-IR data acquisition required just 1 min and 10 μL of serum per sample, and so these results pave the way to fast, specific, and quantitative measurements of protein–drug binding *in vivo* with potentially invaluable applications for the development of novel therapies and personalized medicine.

## Introduction

The ability to measure and understand the interactions between drug molecules and proteins in human blood is important in areas ranging from drug design and biomedical diagnostics to forensic science. The human bloodstream is a complex biomolecular environment containing more than 50 different proteins alongside sugars, phospholipids, and nucleic acids as well as minerals and salts and other metabolites.^1,2^ The potential therefore exists for numerous intermolecular interactions that could influence the behavior and/or efficacy of a drug in the body, which are hard to replicate in the laboratory.

Human serum albumin (HSA), the most abundant of the serum proteins, plays a key role in the binding of drugs to bloodstream proteins. Binding to HSA influences bioactivity by regulating the free drug concentration, which is responsible for pharmaceutical activity as well as modulating the rates of both distribution and excretion.^3^ Serum protein interactions have been identified as the primary contributory factor to the 4% overall success rate for drug candidate development,^4^ which is ascribed to the poor predictability of drug metabolism and pharmacokinetics (DMPK) models due to a reliance on data derived from experimental systems that do not reflect the complexity of the human body.^4,5^

This complexity can be exemplified by considering HSA, which has two main drug binding sites, the properties of which are modulated allosterically by seven fatty-acid binding sites.^6^ Protein–drug interactions are influenced by blood pH, temperature, the presence of other drugs and metabolites, as well as the disease state of the patient, leading to significant inter- and intrapatient variability.^7^ Knowledge of the HSA-bound drug fraction provides valuable insight into the total amount of drug present as well as the potential for HSA to act as a reservoir, maintaining drug levels over long periods, which is also particularly important under conditions of patient overdose.^7−9^ Overall, these factors not only show why it is difficult to predict the *in vivo* behavior of a drug candidate using *in vitro* models but also highlight the value of real-time information in guiding patient treatment. Given that treatment regimens can often feature multiple drugs taken concurrently, the ability to determine the influence of the serum proteins when complex mixtures of drugs are present will allow therapies to be tailored to the individual.^7,9,10^

The current state of the art for measuring serum protein binding *in vivo* uses laboratory-based assays to target either the total drug concentration via protein dissociation or the free fraction only.^11,12^ While delivering good levels of accuracy, these methods feature multiple steps and require long processing times and expensive reagents, adding to experimental costs and delays in obtaining results. In addition, testing for multiple substances via these methods necessitates parallel measurements and many patient samples.^1^

There is thus a requirement at all stages of the development and use of drug molecules for a technique that can be used to responsively measure protein-bound drugs under the complex conditions found *in vivo.* Furthermore, from a fundamental knowledge perspective, although assays deliver information on binding levels, no structural information is obtained. Methods that can provide molecular-level detail of biomolecular interactions in biofluids will also provide vital new data to inform predictive models.

Optical spectroscopies offer routes to fast measurements with minimal sample processing. However, targeting drug binding to HSA *in vivo* presents many technical challenges. Label-free methods are desirable for speed, cost, and efficiency, and the most promising spectroscopic marker for protein-drug binding is the amide I vibrational band, measured by vibrational (infrared (IR) and Raman) spectroscopy. This band reports on protein secondary structure but in IR is obscured by strong, overlapping absorptions of water. For IR, the normal approach of isotope replacement with D_2_O is impractical for biofluid samples. Moreover, IR absorption and Raman methods lack the sensitivity to detect the small changes to the amide I band that occur when drugs bind to HSA in a multiprotein mixture such as serum.^13^

Recently, it has been shown that, unlike IR absorption, two-dimensional infrared spectroscopy (2D-IR) can measure protein amide I signals directly in biofluids by suppressing the water background, conferring the ability to measure protein concentrations and low-molecular-weight fractions of serum optically without sample preprocessing.^13−15^ The nonlinear optical nature of the 2D-IR measurement confers slightly higher frequency resolution of the amide I band via narrower line widths than IR absorption spectroscopy,^13^ and it has been demonstrated that, in contrast to IR absorption methods, amide I band 2D-IR spectroscopy of serum albumin can be used to quantify levels of paracetamol binding at physiological concentrations in equine serum.^16^ Although this is an encouraging result, the promiscuous nature of albumin binding means that key questions remain related to whether the binding signature detected for paracetamol is specific to the identity of the drug molecule or a generic response of the protein to ligand binding and whether the effect is extendable to human serum.

Here we address these important questions by using 2D-IR spectroscopy screening in conjunction with machine learning (ML) techniques. Specifically, we demonstrate classification of four different commonly prescribed drugs, each of which binds to one of the HSA Sudlow sites (I and II), at physiologically relevant concentrations in human serum samples. The implementation of machine learning (ML) algorithms with absorption spectroscopy of biofluids has shown itself to be a valuable tool in the diagnosis of disease.^17−20^ Our analysis shows that each drug generates a unique spectral change to the 2D-IR amide I band of HSA in human serum. This means that the binding of each drug to HSA can be recognized and differentiated while delivering structural insight into the *in vivo* protein–drug interaction. As 2D-IR screening involves a single, rapid (60 s), label-free, low sample volume (10 μL) measurement with no sample preprocessing, this provides proof of concept for an optical tool capable of measuring, differentiating, and quantifying protein–drug binding in a high-throughput manner using *in vivo* sampling, a development that, with further development, we believe has the potential to be of significance to clinical, pharmaceutical, and forensic applications.

## Experimental Section

### Materials

Pooled human serum was obtained from TCS Biosciences. The total HSA content was determined to be 41 mg/mL (0.6 mM) by gel electrophoresis. The four drug molecules, cefazolin sodium, ibuprofen, paracetamol (acetaminophen), and warfarin, were obtained from Sigma-Aldrich and used without further purification.

### 2D-IR Spectroscopy

2D-IR spectra were recorded using the LIFEtime spectrometer at the STFC Central Laser Facility using the Fourier transform time-domain “pump–probe” method based on a sequence of three mid-IR laser pulses (two pump, one probe).^21^ In all cases, the laser pulses were centered at 1650 cm^–1^, with a bandwidth of ∼80 and ∼200 cm^–1^ for the pump and probe pulses, respectively, providing an instrument response time of ∼250 fs. The laser pulse repetition rate was 100 kHz, and the pump pulse pairs with variable pump–pump delay time (τ) required to obtain 2D-IR spectra were produced using a mid-IR pulseshaper.^22^ With averaging, including four-frame phase cycling, the total acquisition time per waiting time (pump–probe delay time, *T*_w_) was 60 s. For each sample, spectra were recorded at *T*_w_ values of 250 fs and 5 ps. The spectrum at *T*_w_ = 250 fs contains protein signals free from the overlapping water band, whereas the spectrum acquired with *T*_w_ = 5 ps contains a thermal water signal that is used in spectral preprocessing (see below).^15,23^

For measurement of 2D-IR spectra, 10 μL of serum (see below for specific sample details) was placed between two CaF_2_ windows without a spacer, and the tightness of the sample holder was adjusted to obtain a consistent absorbance of ∼0.1 for the δ_H–O–H_+υ_libr_ combination mode of water located at 2130 cm^–1^, corresponding to a sample thickness of ∼2.75 μm.^13^ Drug-only control samples (see below) were measured using a sample path length of 25 μm, defined by a PTFE spacer.

### Sample Details

The experiments were divided into three sample sets: the main *2D-IR drug screening study* and two further sample sets named *drug-only control* data set and a *verification* data set. The details of each are described below and summarized in Table 1.

**Table 1:** Summary of Data Sets Used in This Study, Including Samples, Drug Concentrations, and Number of Measurements^a^

2D-IR drug screening data set	molar ratio	molar conc (mM)	conc (μg/mL)	replicates
cefazolin	2:1	1.2	545	3
ibuprofen	2:1	1.2	248	3
paracetamol	2:1	1.2	182	3
warfarin	0.07:1	0.03	10	3
serum	0:1	0	0	12

drug only control data set	molar ratio	molar conc (mM)	conc (mg/mL)	replicates
cefazolin		42	20	1
ibuprofen		97	20	1
paracetamol		133	20	1
warfarin		65	20	1

verification data set	molar ratio	molar conc (mM)	conc (μg/mL)	replicates
cefazolin	10:1	6.0	2900	3
	4:1	2.4	1150	3
	2:1	1.2	572	3
	1:1	0.6	286	3
	0.5:1	0.3	143	3
	0.25:1	0.15	71.4	3
	0.15:1	0.09	42.9	3
	0.1:1	0.03	28.6	3
	0.05:1	0.03	14.3	3
	0.025:1	0.015	7.1	3
	0.01:1	0.006	2.9	3
	0.005:1	0.003	1.4	3
	0.001:1	0.0006	0.3	3
	0:1	0	0	3

a The HSA concentration was determined to be 0.6 mM. Gray headings indicate separate data sets.

#### 2D-IR Drug Screening Study

For the 2D-IR screening study, cefazolin sodium (cefazolin hereafter), ibuprofen, and paracetamol were each added to pooled human serum to give a total drug concentration of 1.2 mM. This produced a constant 2:1 molar ratio relative to HSA and constitutes a clinically relevant concentration in each case, being either within or slightly in excess of the normal dosage range (Table 2).^24−26^ Clinical doses of warfarin are significantly lower than the other three drugs, and so warfarin was spiked into serum at a concentration of 30 μM, giving a molar ratio of 0.07:1 to HSA.^27^ Previously determined binding constants for each drug with HSA are given in Table 2 and span the 0.5–100 μM range, although we note that these were not determined in serum. Each sample was measured in triplicate alongside 12 measurements of neat serum to provide a balanced serum vs serum–drug data set for subsequent analysis (Table 1).

**Table 2:** Typical Clinical Ranges for the Four Drugs Used in This Study Suggest That Overdose Values Exceed This Range^a^

	**clinical range**	**binding constant (*K***_**d**_**)**
cefazolin sodium	0.04–0.5 mM^24^	50 μM^30^
ibuprofen	0.05–0.5 mM^25^	0.5 μM^31^
paracetamol	0.2–1.3 mM^26^	100 μM^32^
warfarin	2–8 μM^27^	3 μM^33^

a Associated HSA binding constants (*K*_d_) are also shown.

#### Drug-Only Control Data Set

Solutions of each of the four drugs in DMSO were prepared to allow measurement of the spectral contributions of each in the amide I region of the spectrum. Concentrations of 20 mg/mL were used in each case (Table 1), which are significantly higher than in the screening study to ensure strong 2D-IR signals. DMSO was chosen as a common solvent as ibuprofen exhibits poor solubility in water, ∼20 mg/L (∼0.1 mM).^28^

#### Verification Data Set

A verification sample set was used to test the outcome of the screening study. A stock solution of cefazolin in human pooled serum at a concentration of 2.9 mg/mL (6 mM; 10:1 molar ratio with HSA) was sequentially diluted with pooled serum to create 13 samples with cefazolin concentrations of 6 mM–0.6 μM plus neat serum, spanning the clinically relevant range (Table 1).^24^ The most dilute sample corresponded to a drug content of just ∼300 ng/mL. Each sample was measured in triplicate.

### Data Preprocessing

After 2D-IR data collection, spectral preprocessing was carried out for all serum samples using a previously described workflow designed to enable reliable cross-comparison of spectra obtained from different samples.^15^ Briefly, the small thermal response of the water component of the samples measured using a *T*_w_ of 5 ps was used to perform a bandwidth-guided baseline correction and normalization to account for instrumental and sample path-length variations between measurements.^23^ Savitsky–Golay smoothing and principal component analysis noise reduction were also performed.^15^ All data preprocessing and subsequent analysis were carried out using custom scripts written using R.^29^

### Data Analysis

#### 2D-IR Drug Screening Study

Preprocessed 2D-IR spectra were analyzed via partial least-squares discriminant analysis (PLS-DA) using the R package *caret* to classify the data into relevant groupings. PLS-DA is a supervised multivariate dimension reduction technique that identifies latent variables (LVs), which maximize the covariance between the input spectral data set and its class labeling.^34,35^ The first LV contains the maximum covariance between the original data set and its labeling, with subsequent LVs containing progressively less significant information. The number of LVs and mean-centered parameters were examined, and the highest performing results are discussed here, which include no mean-centering. The 2D-IR spectra of all 24 samples (12 neat serum and 12 containing one of the four drug molecules) were initially subject to binary classification, using the labels “Serum” and “Drug bound”, where “Serum” is pooled human serum with no additives and the latter collects all samples with one of the four drugs added to the human serum as a single “Drug bound” faction. Thereafter, a five-category classifier was applied with the following labels: “Serum” (pooled human serum) and “Serum and Cefazolin”, “Serum and Ibuprofen”, ‘Serum and Paracetamol”, and “Serum and Warfarin” denoting samples containing serum spiked with the respective drug.

## Results and Discussion

**Figure 1:**
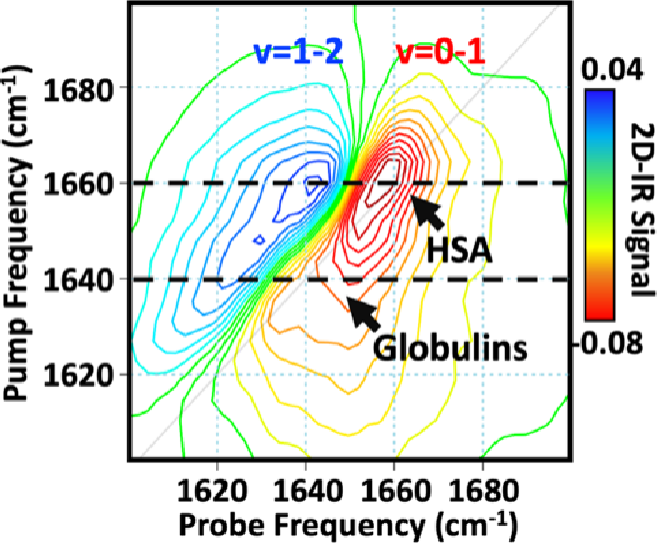
Preprocessed 2D-IR spectrum of pooled human serum. Dashed black lines at pump wavenumbers of 1660 and 1640 cm^–1^ indicate peak positions of HSA and globulins, respectively. The *v* = 0−1 and *v* = 1−2 vibrational transitions of the amide I bands are shown in red and blue respectively.

The 2D-IR spectrum of a sample of human pooled serum in the protein amide I region of the infrared shows two bands (Figure 1). A negative band (red) located on the spectrum diagonal (pump = probe = 1660 cm^–1^) is due to the fundamental, *v* = 0–1, transition of the protein amide I mode (showing loss of vibrational ground-state population and stimulated emission from *v* = 1–0 caused by the pump). A positive (blue) peak due to the *v* = 1–2 transition of the same mode (showing the population of the *v* = 1 state) is shifted to a lower probe wavenumber due to anharmonicity.

In principle, these two bands contain contributions from all proteins in the serum sample. However, HSA is the most abundant serum protein and so dominates the spectrum near 1660 cm^–1^ as a result of its largely α-helical structure (Figure 1).^13^ A smaller feature near 1640 cm^–1^ is assignable to the globulin protein group that constitutes the nonalbumin fraction, reflecting the β-sheet rich structures of the globulins.^13,36^

### 2D-IR Drug Screening Study

**Figure 2:**
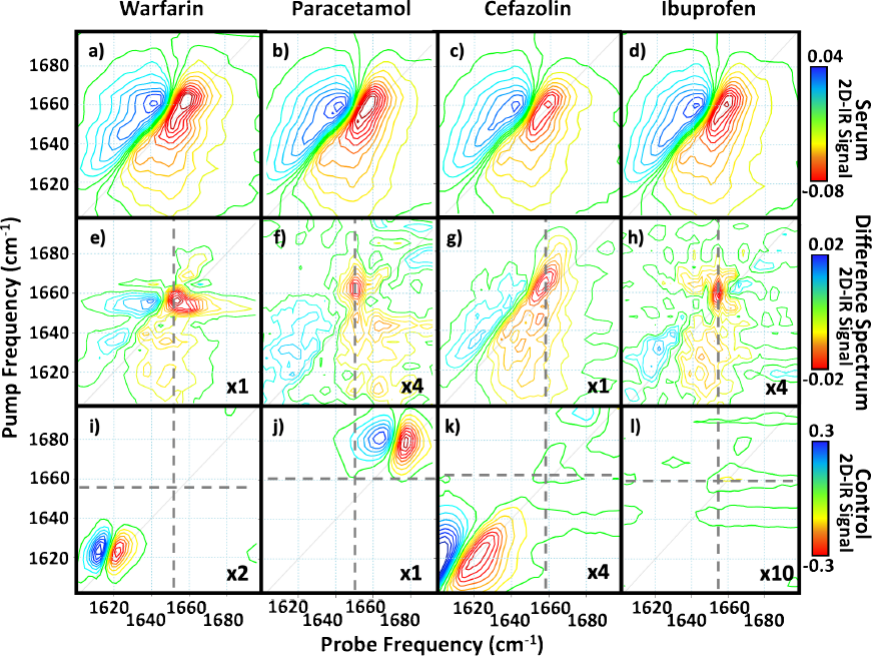
(a–d) Preprocessed 2D-IR spectra of human serum with the addition of (a) warfarin, (b) paracetamol, (c) cefazolin, and (d) ibuprofen at the concentrations shown in Table 1. (e–h) 2D-IR difference spectra obtained via subtraction of a neat serum spectrum (Figure 1) from the spectra in panels a–d): (e) warfarin, (f) paracetamol, (g) cefazolin and (h) ibuprofen. (i–l) 2D-IR spectra of the drug-only control data set, (i) warfarin, (j) paracetamol, (k) cefazolin, and (l) ibuprofen, each at a concentration of 20 mg/mL in DMSO, measured using a 25 μm spacer. Spectra are plotted on scales shown for each row and magnified by the factor indicated in the bottom right-hand corner. Gray dashed lines denote difference spectrum peak position for each drug. *T*_w_ in all cases was 250 fs.

The 2D-IR spectra of the serum samples following the addition of cefazolin, ibuprofen, paracetamol, or warfarin (Figure 2a–d) show subtle differences from the neat serum spectrum (Figure 1). These differences are highlighted by subtracting the spectrum of neat serum from each of the drug-added samples (Figure 2e–h, Figure S1). We confirm that these differences do not arise from vibrational modes of the free drug molecules themselves by comparison with the 2D-IR spectra of the drug-only control samples (Figure 2i–l). It is noted that although the drug-only control samples were measured using DMSO, the wavenumber separations of the drug-derived signals (Figure 2i–l) from those of the serum proteins (Figure 2e–h) mean that any solvent-derived shifts will be insufficient to implicate the drugs as the cause of the difference spectral signals. In addition, the drug-only control samples were obtained using an order of magnitude higher concentration and a further order of magnitude greater path length than the serum samples. As such, any contributions from the drug molecules will be vanishingly small.

### Identification of Bound Drugs

**Figure 3:**
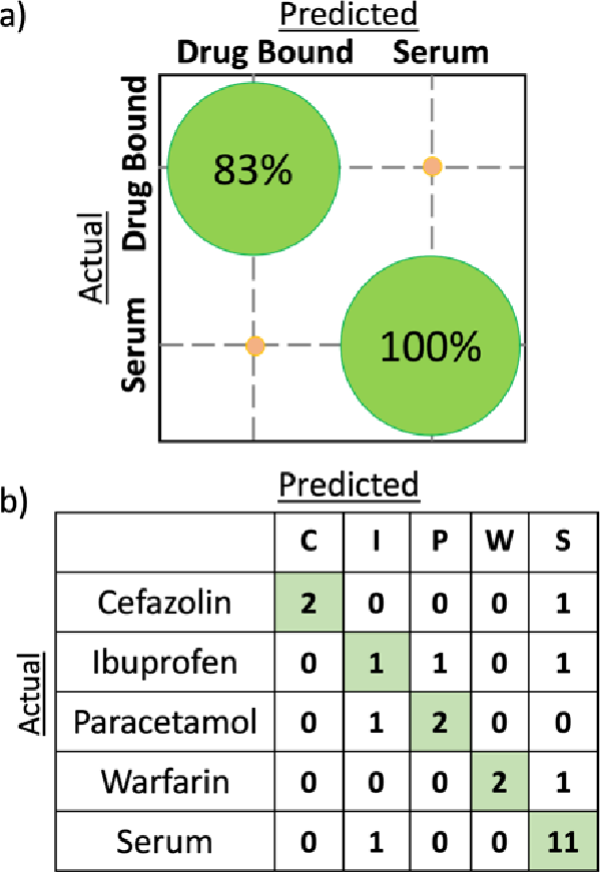
Model predictions using leave-one-out cross-validation with partial least-squares discriminant analysis. (a) The binary model (using seven LVs) confusion ball demonstrating correct classification of all 12 (100%) serum samples and 10/12 (83%) drug-bound serum samples. (b) The confusion matrix for the five-class model (using nine LVs) highlights the number of samples correctly identified (table diagonal, green).

The aim of the study was to determine whether 2D-IR spectroscopy combined with ML methods can be used to identify the presence of drugs bound to HSA in human serum and to spectroscopically differentiate between them. Initially, a binary PLS-DA model was implemented to deliver classification between neat serum and those containing drugs but with no differentiation between drug types attempted. The PLS-DA machine learning method produces a model built using data that is catalogued as the “training data set”. In the binary example, the model aims to identify differences between “Serum” and “Drug-bound” sample classes, of which there were 12 of each in this study (Table 1). To assess the predictive ability of the model, a leave-one-out cross-validation (LOO-CV) protocol was used, which is the typical approach for small data sets^35^ and involves removing each sample from the training data set in turn and re-evaluating the predicted classification using the data from the remaining 23 samples. A binary model using seven LVs was found to correctly identify all serum samples and 10 of the 12 drug-bound samples (Figure 3a), yielding a total model accuracy of 92%.

### Differentiation of Bound Drug

Although the binary classification approach produces high accuracy, consistent with the fact that changes to the serum spectrum could be observed using a traditional difference spectrum approach (Figure 2e–h), it does not allow differentiation between the effects of individual drugs. To address this, a more complex PLS-DA model containing five classes and specific drug labeling was applied. The five-class model follows the same format as the binary model, including LOO-CV. The confusion matrix for the five-class model using nine LVs (Figure 3b) displays the number of each sample type correctly identified along the diagonal (green), whereas unsuccessful predictions appear off the diagonal. Of the 12 serum samples, 11 (92%) were correctly identified along with 7 of the 12 drug-containing samples. Samples containing cefazolin, paracetamol, and warfarin were correctly identified in two out of three cases (67%), whereas the success level for ibuprofen (33%) was lower. An overall model accuracy of 75% (18 of 24 samples), however, provides proof-of-principle for drug classification using 2D-IR spectra.

### Linking Classification to Protein Structure

We now turn to discuss the direct link between the 2D-IR amide I spectral maps and protein secondary structure and dynamics in terms of the spectral variations revealed by the PLS-DA model, which contains molecular information about the impact of drug binding on the serum proteins. This information is encapsulated within the LVs on which the five-class model bases classification decisions. These are represented as *loadings,* a set of spectral amplitudes at each pump–probe coordinate that comprise 2D-IR spectral maps with regions of high variance associated with that LV. The accompanying *score* shows the contribution of the LV loading to each sample. If specific LVs are strongly associated with a particular sample classification (serum or each of the four drugs), this would provide direct visualization of the spectral changes linked to each sample type.

**Figure 4:**
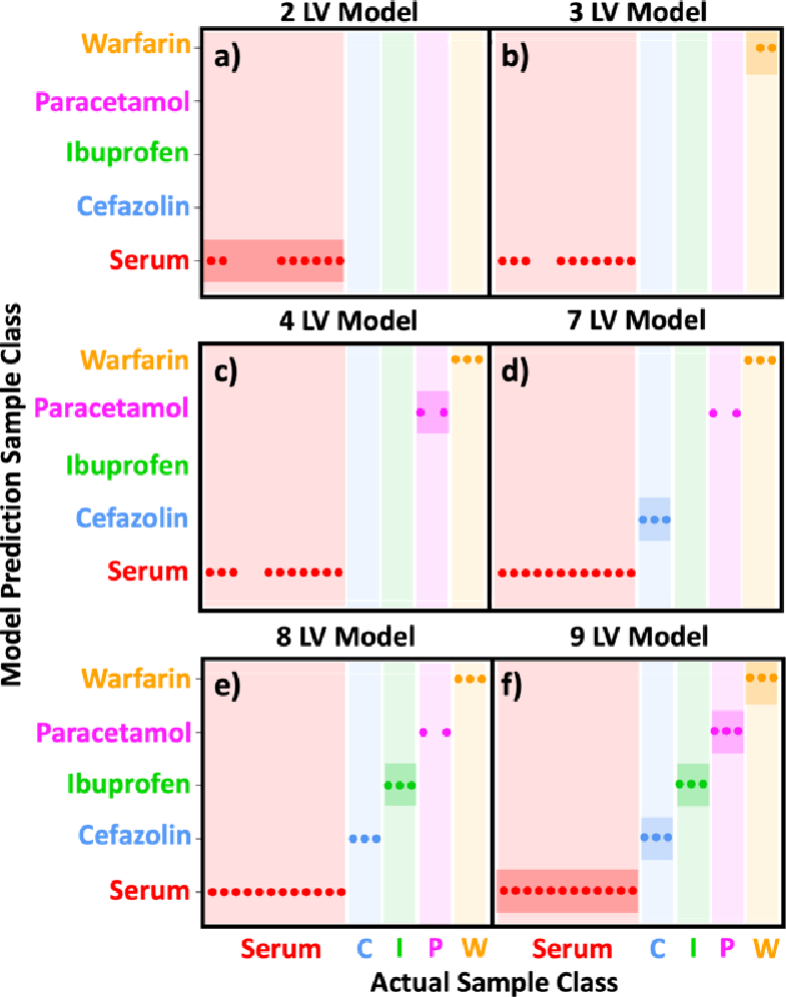
Results of a five-class PLS-DA model using up to nine latent variables (a–f). Each panel shows the classification success for each class (serum only (red), warfarin (orange), paracetamol (pink), ibuprofen (green), and cefazolin sodium (blue)). Dots indicate successful validations.

Using a PLS-DA model containing all 24 samples, the number of LVs included was increased incrementally (Figure 4). The results show that inclusion of the first two LVs provided sufficient spectral information to categorize the majority of the serum samples (67%, Figure 4a). Introduction of the third LV enabled the model to identify 67% of the samples containing warfarin (Figure 4b), whereas the fourth, seventh, and eighth LVs allowed classification of the paracetamol-, cefazolin-, and ibuprofen-containing samples, respectively (Figure 4c–e). A nine-LV model provided enough information to classify all samples (Figure 4f, Figures S2–3).

Although a low number of LVs are desirable to avoid introducing unnecessary bias into the model,^37^ our data set contains five categories, and the nature of the binding interactions means that there may be spectral changes shared between drugs whereas others may be drug-specific. The use of nine LVs is therefore realistic and was found to coincide with the elbow in the root-mean-square error (RMSE) plot for the cross-validation predictions (Figure S4).

**Figure 5:**
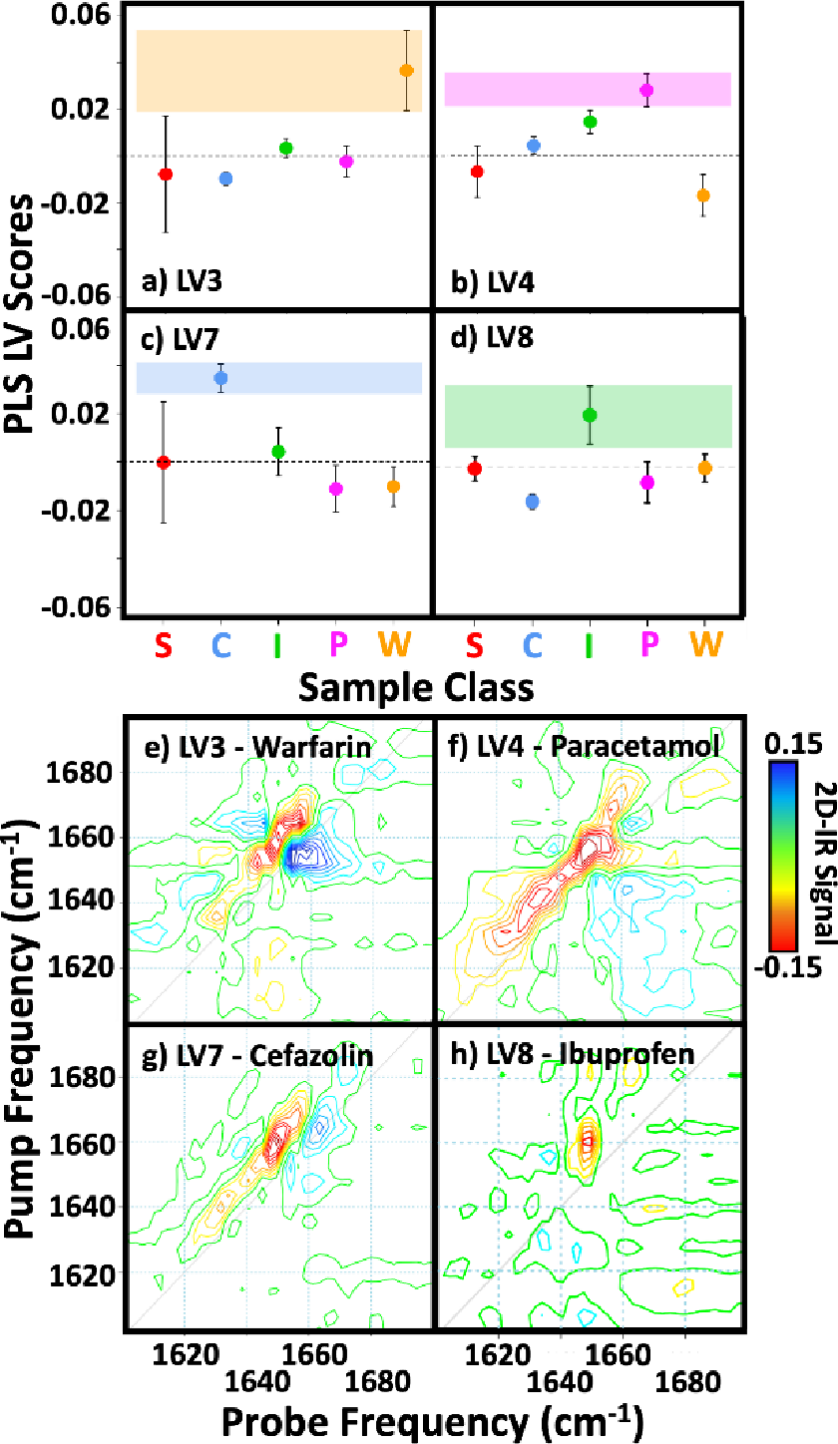
Scores (a–d) and loadings (e–h) for latent variables 3, 4, 7, and 8 allowing sample categorization using the partial least-squares discrimination analysis model described in Figure 4. The scores highlight the weighting of the loading for each drug. Each point is color coded (cefazolin (blue), ibuprofen (green), paracetamol (pink), warfarin (orange), and serum (red)) and denotes the mean score (*n* = 3 for all drug samples and *n* = 12 for neat serum). Error bars are 1σ. Black dotted horizontal lines in panels a–d denote a score value of 0. The loadings display the 2D-IR lineshapes and structural variations related to each drug.

This analysis suggests that certain LV loadings can be used to describe the main spectral features associated with the presence of individual drugs with confidence. Considering the relative contributions (scores) of each LV to the spectra of the four drug-containing samples (Figure 5a–d) confirms this result.

The average scores for each LV and sample class are shown with 1σ variation indicated as error bars. Figure 5a shows that the warfarin samples (orange) have the highest scores for LV3, with the LV3 scores of the other drug samples scattered around zero. Significantly, there is no overlap of the warfarin LV3 score with that of any other sample class (Figure 5a, orange bar). This is consistent with the qualitative result that the addition of LV3 enabled classification of the warfarin samples (Figure 4b). Similar conclusions apply to the other three drugs: paracetamol (pink, Figure 5b), cefazolin (blue, Figure 5c), and ibuprofen (green, Figure 5d), which have the largest scores for LVs 4, 7, and 8, respectively, emphasizing that drug-specific 2D-IR spectral patterns are observed in each case. It is noted however that some correlation exists between LVs. For example, adding LV4 to the model slightly improves the categorization of warfarin while also significantly improving that of paracetamol.

Examining the LV loadings associated with each drug by this process (Figure 5e–h) shows that each plot contains spectral features close to the spectrum diagonal, concentrated around 1660 cm^–1^. This region of the serum amide I spectrum is principally assignable to HSA, indicating that the features are due to drug binding to HSA, consistent with reported *K*_d_ values (Table 2).^7,30−33^

### Verifying the PLS-DA Drug Specificity

The LVs determined by the PLS-DA model can be used to differentiate the spectra of the samples, but a question remains as to whether these effects offer repeatable physical spectral insight or a model-specific outcome. To address this, a verification data set was created in which cefazolin was added to serum at a range of concentrations from 0.6 μM to 6 mM (Table 1). The resulting 2D-IR spectra were preprocessed in the same way, but a different analysis method using PLS regression (PLS-R) analysis was applied. No spectra or samples were common to the verification and screening experiments. In this case, PLS-R also leads to calculation of LVs with loadings and scores, but the spectral covariances will be maximized as a function of the cefazolin concentration.

**Figure 6:**
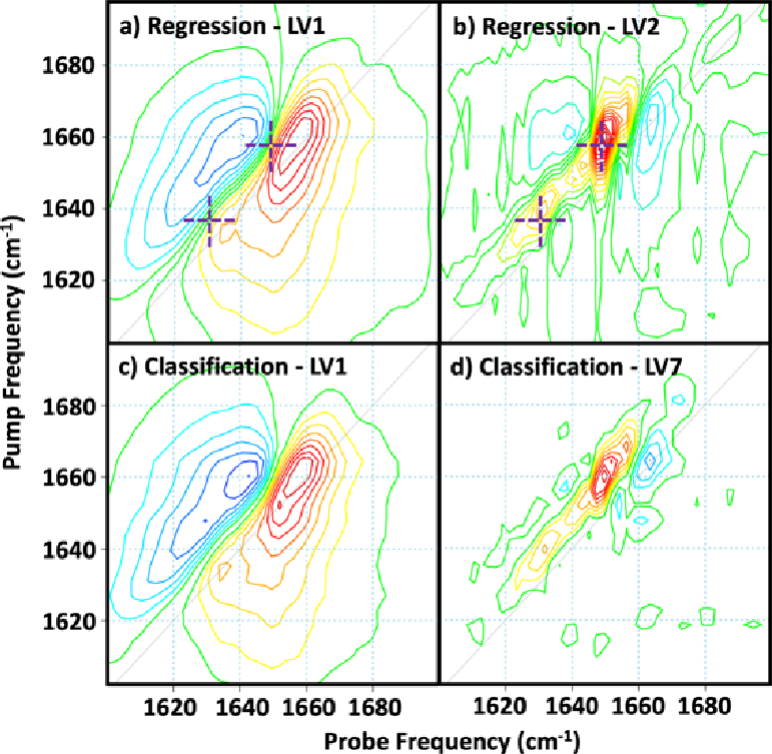
Comparison of latent variable (LV) spectral loadings produced from screening and verification data sets. Verification: (a) LV1 shows the average spectrum, and (b) LV2 shows the spectral variation as a result of cefazolin binding. Crosshairs mark data points used to quantify cefazolin concentration at pump and probe frequencies ((1635, 1632 cm^–1^) and (1656, 1649 cm^–1^)). Screening: Results from the serum drug binding classification analysis showing (c) LV1 “serum” and (d) LV7 demonstrate the changes associated with cefazolin binding.

The resulting PLS-R loading plots (Figure 6a,b) show that LV1 is very similar to the neat serum spectrum, as expected, whereas LV2 shows the dominant spectral change associated with the cefazolin presence. Comparing the results of the PLS-R validation study with the original classification model result for cefazolin (Figure 6c,d), which identified cefazolin with LV7, shows excellent agreement. Obtaining the same result via two different studies thus confirms that the LVs can be assigned to genuine spectral changes.

**Figure 7:**
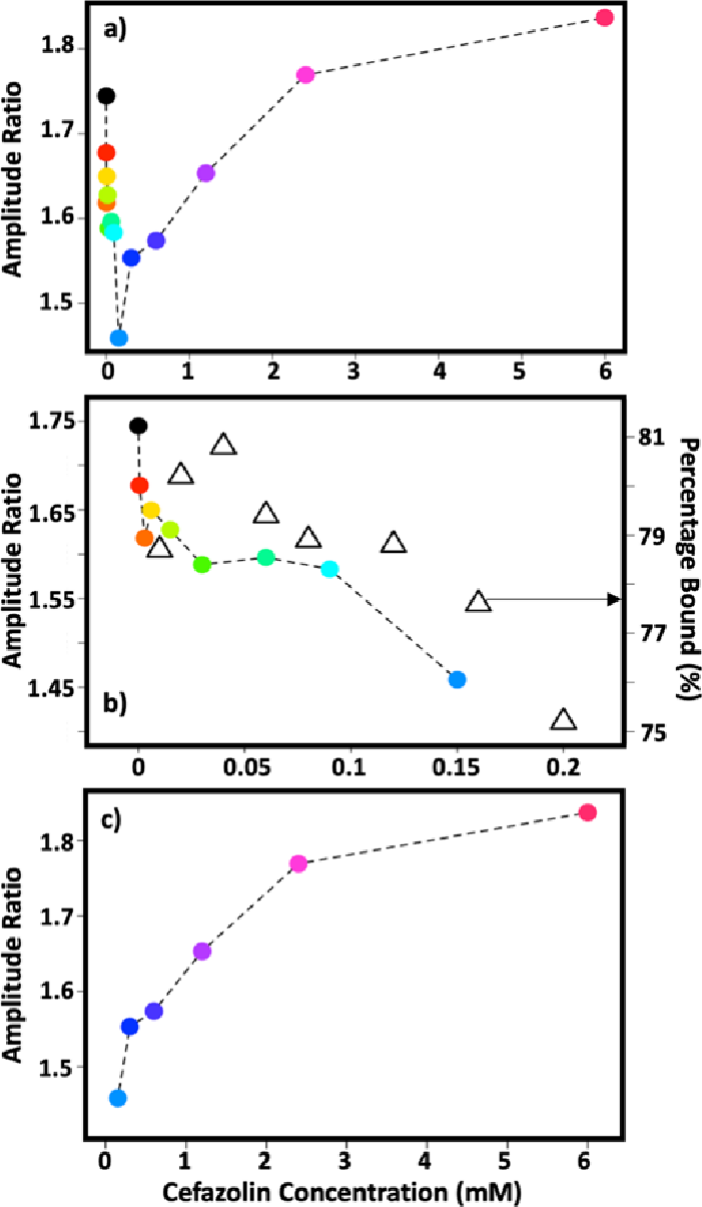
Amplitude ratio of 2D-IR spectra at points shown in Figure 6a,b as a function of cefazolin concentration. The ratio compares the high frequency point (1656, 1649 cm^–1^) to the lower frequency peak (1635, 1632 cm^–1^). (a) Amplitude ratio as a function of the full cefazolin concentration range studied. (b) The same results focusing on the 0–0.2 mM range. Hollow triangles show the percentage of cefazolin bound to HSA in serum as a function of cefazolin concentration from ref (30). (c) Amplitude ratio of samples containing more than 0.2 mM of cefazolin.

Examining the dependence of LV2 upon cefazolin concentration revealed an unusual U-shaped profile involving a rapid decrease in LV2 score up to a cefazolin concentration of 150 μM before the score increased toward an apparently limiting value at very high (mM) concentrations (Figure S5). Using LV2 as a guide to the key changes in spectroscopy occurring upon cefazolin addition, we examined the 2D-IR spectral amplitude ratio at the LV2 maxima identified in Figure 6b (crosses) and found this to show a similar behavior with cefazolin concentration (Figure 7a).

Although the LV2 score and amplitude ratio would be expected to correlate with cefazolin binding to HSA in our experiments, such a U-shaped profile is clearly inconsistent with a simple model of protein binding. However, previous studies of cefazolin interactions with serum have reported that cefazolin shows dose-dependent binding leading to a reduction in bound fraction at concentrations exceeding 200 μM.^30^ Indeed, comparing the cefazolin concentration dependence of the spectral amplitude ratio identified via LV2 (Figure 7b, colored dots) with the measured bound percentage of cefazolin directly from ref (30) (Figure 7b, open triangles)^30^ shows remarkable agreement below 200 μM. As the two sets of experiments were performed under near-identical conditions with respect to HSA and cefazolin concentrations, we thus conclude that our results correlate with the cefazolin bound fraction. It is noteworthy that some variation from the published data occurs at very low (a few μM) cefazolin concentrations, which may indicate the limit of detection of 2D-IR in this case. Beyond 200 μM, our data show a reverse trend (Figure 7c) consistent with the observed reduction in bound percentage,^30^ although the concentration range studied here is wider than that reported previously, which did not exceed 300 μM.

Overall, this suggests that, in addition to identifying the presence of HSA-bound cefazolin in serum, the 2D-IR-derived data in Figure 7a can be used to quantify the binding behavior of cefazolin to serum proteins. This extends the findings of a previous study in which we used 2D-IR to quantify the level of paracetamol bound to equine serum albumin.^16^ Our new data show, in agreement with previous work, that cefazolin–serum interactions can be separated into two regimes. At low concentrations (sub 200 μM), binding to HSA follows a normal behavior before a second mechanism at higher cefazolin concentrations leads to what appears to be a reduction in HSA affinity and the U-shaped profile observed. Such atypical behavior is well-recognized in pharmacokinetics and dynamics studies and is believed to arise from the complex molecular environment of serum.^38^ The specific mechanisms leading to atypical binding profiles vary between drugs, but roles for fatty acid binding, the involvement of secondary binding sites at high drug concentrations, or changes in ionic concentrations have all been implicated. Although the origins of the trend are unclear in this case,^38^ the addition of spectroscopic information from 2D-IR shows that, as well as the HSA-centered feature near 1660 cm^–1^, weaker features located at lower pump frequencies are observed in both the screening study (LV7) and the verification study (LV2) (Figure 6b,d). This secondary feature lies in the globulin region of the amide I response of serum and so may indicate cefazolin interaction with proteins in serum other than HSA. One possibility is α1-acid-glycoprotein, which has been shown to bind cefazolin^39^ and has predominantly a β-sheet secondary structure;^40^ this would be expected to give rise to an amide I band near 1640 cm^–1^ in H_2_O rich media.^13,36^ Thus, it is plausible that the secondary binding mechanism may involve other serum proteins, although more work will be required to fully understand the molecular origins of the binding profile.

A major benefit of using this 2D-IR approach to study binding is that the LV loadings can be used as a guide to identify key changes caused to HSA by drug binding in each of the four cases. We now turn to discuss several broad interferences drawn from the classification data set.

First, it is noteworthy that each drug molecule elicits a different spectral response from HSA, showing a unique impact upon either the secondary structure or dynamics of the protein upon binding (Figure 5e–h). The plots are broadly comparable with the difference spectra obtained upon drug addition in that the largest features are consistent (Figure 2e–h and Figure S6), particularly so for cefazolin and ibuprofen. However, as the PLS-DA model identifies the *principal* sources of spectral variance for each drug, it should not necessarily follow that the loadings will be identical to the difference spectra. This highlights the different applications open to 2D-IR studies of protein drug binding, ranging from an analytical measure of drug binding and classification, using ML methods as we show here, to a detailed spectroscopic study of structure change upon drug binding, which can be achieved via analysis of the difference spectral response. The latter will however require more detailed study to fully associate the information from the 2D spectra with structural effects of binding. Using the LV loadings as a guide to the key changes caused to HSA by drug binding in each of the four cases, broad inferences can however be drawn. The binding of warfarin and cefazolin shows similar patterns of negative and positive changes in peak amplitudes, though at slightly different frequencies. These changes could indicate a reduction in the intensities of the amide I components from α-helix structures in HSA upon binding, perhaps indicative of a reduction in coupling due to distortion or a change in the relative orientations of helical segments of the structure.^41^ Interaction of drug molecules with HSA is known to occur primarily through one of two binding sites (Sudlow I and II), and warfarin and cefazolin are both known to bind to site I, which may account for the similar structural impact.^30,33^

A common binding site may explain the similar spectral changes associated with warfarin and cefazolin, although this argument does not extend to paracetamol and ibuprofen that target site II.^31,32^ Ibuprofen shows a peak located on the low-frequency side of the spectrum diagonal. Such an observation has been associated with proteins becoming less dynamic upon ligand binding, leading to changes in anharmonicity.^7,8^ By contrast, the impact of paracetamol binding to HSA yields a spectral change over an elongated frequency range. This is consistent with a recent study of paracetamol binding in equine serum and assigned also to anharmonicity changes.^16^ However, such spectral changes seen here may also be due to alterations of the diagonal line width.^15^

With the associated spectral changes of each of the four drugs being unique, we can conclude that each drug engages with HSA differently and cannot be simply identified via one of the two Sudlow sites. Even if the spectral changes are broadly similar, as with warfarin and cefazolin, the spectral changes vary in magnitude and absolute shape, suggesting that the structural effects may follow a continuum rather than a simple “on/off” model. Further effects will arise from the impact of fatty acid binding sites, and the competitive nature of binding to HSA may well contribute to differences observed, and this study will motivate more detailed investigation of such effects to exploit the full potential of the information contained within the spectra.

## Conclusions

These results constitute the first direct optical differentiation of drugs binding to HSA in human biofluids. With continued development, the ability of 2D-IR to characterize the unique spectral impacts of binding four different drugs to HSA has the potential to be useful from both clinical and pharmaceutical perspectives. Detailed knowledge of specific drug-binding interactions has the potential to aid drug development via the delivery of structural information while offering a route to measuring the kinetics and dynamics of drugs *in vivo.* The ability to positively differentiate between drugs binding to HSA via spectroscopic signatures also offers the scope for quantitative measurement in complex situations where multiple drugs are present, enabling drug–drug interference to be measured and opening up applications in personalized treatment management. Of particular practical benefit for applications of machine learning such as this would be the collection of larger data libraries, which would be expected to bring improvements in predictability and accuracy. In the case of *in vivo* studies, the ability that 2D-IR provides in terms of acquiring these data quickly and with minimal manipulation following sample collection will also be beneficial in terms of repeatability and accuracy.

The study further illustrates that drug-specific conformational changes can be identified within the HSA protein upon drug binding, highlighting the sensitivity and repeatability of 2D-IR to detect drug binding in native solutions while providing insight that can be linked via the 2D map to protein structure and dynamics. In addition, these unique features have the capability to provide quantitative results relating to bound drug concentrations and further demonstrate the usefulness of the amide I band to detect and “enhance” the drug signal, which would otherwise be too weak to detect.

Although our results fully justify further work to understand the full extent of the information available via the more subtle effects observed in the 2D-IR signatures, the evidence we present shows that binding to HSA is not a straightforward interaction, with different drugs binding differently to the protein molecule.

The method can also be considered to be extendable to other protein–drug combinations, and more detailed parametrization of spectral responses would provide valuable metrics for use during 2D-IR screening of drug candidates binding to target proteins. We believe that these findings provide the foundations for progression to the use of larger spectral data sets to improve the quality of ML models toward a fundamental understanding of protein amide I spectroscopy in H_2_O-rich fluids. Ideally, this would also fuel theoretical engagement to model the spectroscopic outcomes and relate the details of the protein structural changes revealed by the LV loading plots.

The application of 2D-IR, when combined with our methodologies for analysis in aqueous solutions, highlights significant progress toward both detection and further understanding of drug–protein complexes. Although variations in protein structure upon binding have been observed using other analytical methods, 2D-IR is able to provide information under physiological conditions using small volumes of as-received samples in a short time span. The combination of binding data with structural insight and speed places 2D-IR in a niche between fast methods, which reveal binding but with no structural data (e.g., surface plasmon resonance), and slower methods, which provide atomistic structural insight but require extensive sample preparation as well as data collection and analysis time, making 2D-IR a viable analytical method from both a simplicity and economic standpoint.

## Supporting Information Available

Additional 2D-IR spectra and complete output of the PLS-DA analysis (PDF)
