# Supplementary material for: Optical Screening and Classification of Drug Binding to Proteins in Human Blood Serum
Source: Anal Chem. 2023 Nov 8;95(46):17037–45. doi: 10.1021/acs.analchem.3c03713 (PMC10666086; doi:10.1021/acs.analchem.3c03713)
Supplement: Supplementary file 1 — ac3c03713_si_001.pdf [file ac3c03713_si_001.pdf]

Supporting Information

Optical Screening and Classification of Drug-binding to Proteins in Human Blood Serum

Samantha H. Rutherford,[a] Christopher Hutchison,[b] Gregory M. Greetham,[b] Anthony W. Parker,[b] Alison Nordon,[c] Matthew J. Baker, [d] and Neil T. Hunt.*[e]

[a.] *WestCHEM, Department. of Pure and Applied Chemistry, University of Strathclyde, Technology and Innovation Centre, 99 George Street, Glasgow Glasgow, G1 1RD, UK*
[b.] *STFC Central Laser Facility, Research Complex at Harwell, Rutherford Appleton Laboratory, Harwell Campus, Didcot, OX11 0QX, UK*
[c.] *WestCHEM, Department. of Pure and Applied Chemistry and CPACT, University of Strathclyde, 295 Cathedral Street, Glasgow, G1 1XL, UK*
[d.] *School of Medicine and Dentistry, University of Central Lancashire, Fylde Rd, Preston, PR1 2HE, UK*
[e.] *Department of Chemistry and York Biomedical Research Institute, University of York, Heslington, York, YO10 5DD, UK*

Table of Contents

Figure S1……………………………………………………………………………………S2

Figure S2……………………………………………………………………………………S2

Figure S3……………………………………………………………………………………S3

Partial least squares – discriminant analysis……………………………………………..………S3

Figure S4……………………………………………………………………………………S4

Figure S5……………………………………………………………………………………S4

Figure S6……………………………………………………………………………………S5

References…………………………………………………………………………………..S5

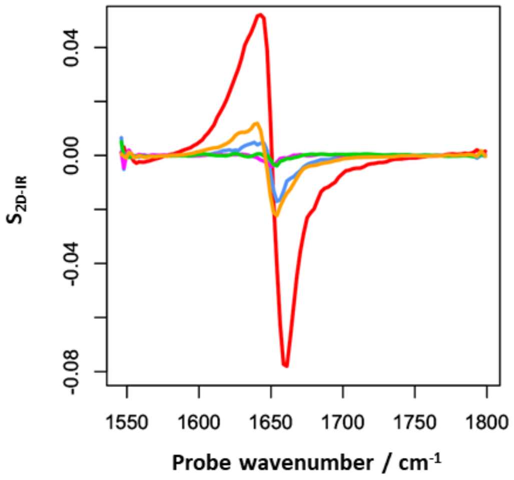

Figure S1: *Comparison of slices through 2D-IR spectra from Figure 2. The red trace shows a cut through the spectrum of a neat serum sample at a pump wavenumber of 1660 cm^{-1}. The colored traces are similar slices through the difference spectra obtained upon addition of each drug (Fig. 2(e-h)): Cefazolin (blue), Ibuprofen (green), Paracetamol (pink), Warfarin (yellow).*

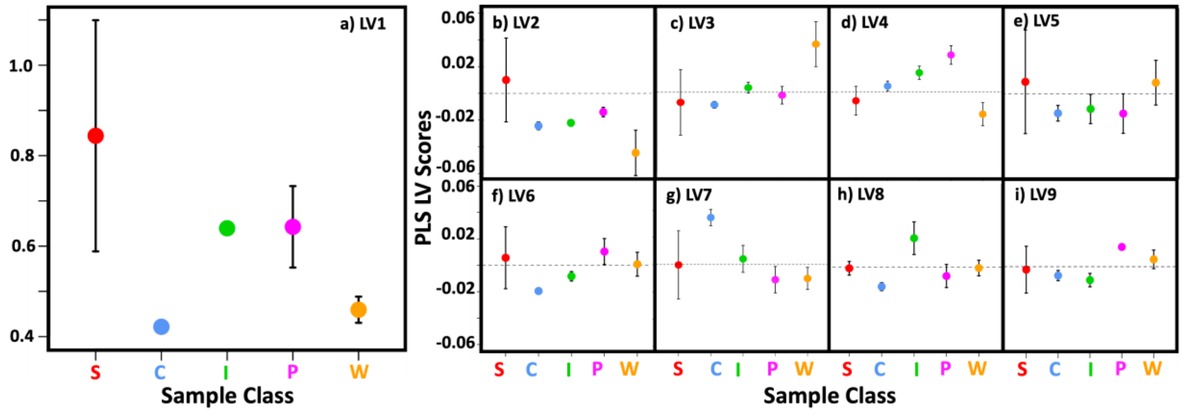

Figure S2: *Scores for all latent variables 1 through 9 following PLS-DA model described in main text: a) LV1, b) LV2, c) LV3, d) LV4, e) LV5, f) LV6, g) LV7, h) LV8, i) LV9. The scores highlight the weighting of the loading for each drug. Each point is color coded: cefazolin (blue), ibuprofen (green), paracetamol (pink), warfarin (orange) and serum (red); and denotes the mean score (n=3 for all drug samples and n=12 for neat serum). Error bars are 1σ. Black dotted horizontal lines in denotes a score value of 0.*

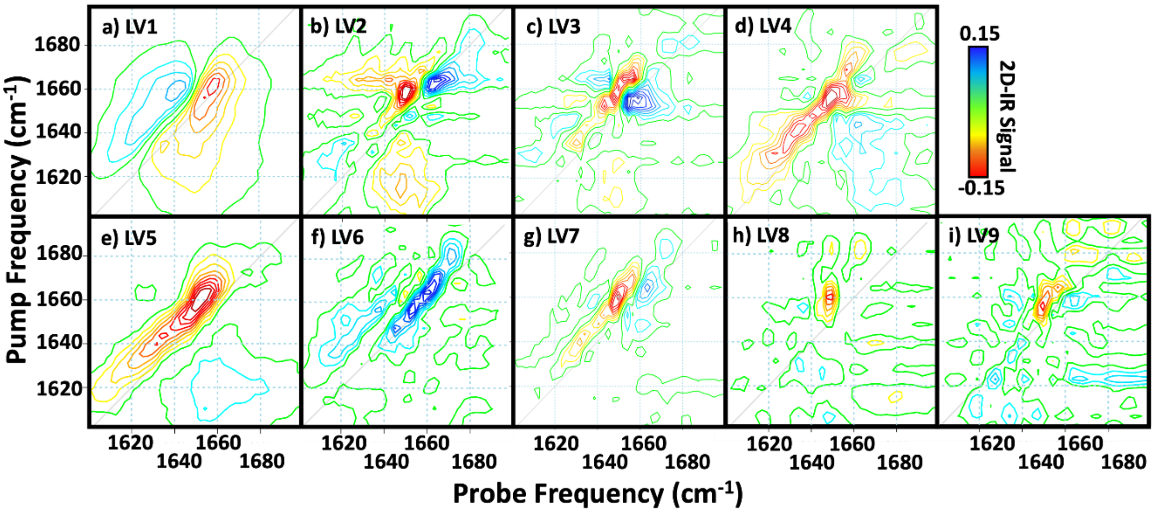

Figure S3: *Loadings for all latent variables (LV) 1 through 9 following partial least squares – discrimination analysis model described in main text: a) LV1, b) LV2, c) LV3, d) LV4, e) LV5, f) LV6, g) LV7, h) LV8, i) LV9.*

Partial least squares – discriminant analysis

The optimum number of latent variables (LVs) for the partial-least squares-discriminant analysis (PLS-DA) models was selected based on two criteria: the root mean square error (RMSE) of cross validation (CV) and the CV model accuracy. While a low number of LVs are desirable to avoid introducing unnecessary bias into the model,[1] consideration of what the dataset contains and the expected number of LVs is crucial. For example, the use of two LVs is not appropriate when trying to model a dataset containing 4 drug interactions and a control. If specific drug interactions are to be extracted, the number of LVs selected should reflect this. Thus before any analysis on LV selection is performed, the minimum number of LV must be 5, to allow for a single LV for each class.

Analysis of the leave one out cross validation results provides a more comprehensive approach to the selection of LVs, for both the binary and multiclass models. By splitting the model from 24 samples to 23 samples and 1 test, we can iterate across each sample and assess the predictability of the model using the current dataset when additional blind test sets are not available. Examination of the RMSE of the CV results, is an absolute measure of error between the observed and predicted values, with lower values indicating higher predictive powers. For both the binary and multiclass models (Fig. S2(a,c)) the RMSE decreases with an increase in the number of LVs, with 7 and 11 LVs providing the smallest RMSE of CV, respectively. The CV prediction accuracy is also shown (Fig. S2(b,d)) and demonstrates that the binary and multiclass models achieve maximum accuracies of 92 % and 75% using 7 and 9 LVs, respectively.

Thus, for the binary model a total of 7 LVs were selected. As for the multiclass model, we have either 11 LVs from the RMSE-CV or 9 from the CV Accuracy. As lower numbers of LVs are typically desirable to prevent introducing unnecessary bias into the model,[1] 9 LVs were selected for the multiclass model.

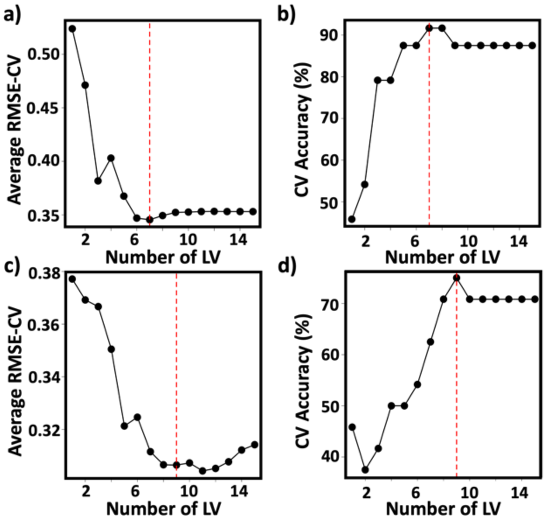

Figure S4: *Measures of partial least squares – discriminant analysis (PLS-DA) cross validation (CV) performance with the number of latent variables, for the binary model (a,b) and the multiclass model described in the main text (c,d). (a,c) gives the root mean square error (RMSE) of CV and (b,d) highlights the CV prediction accuracies. Red dashed vertical lines denote 7 LVs (a,b) and 9 LVs (c,d) for the binary and multiclass models, respectively.*

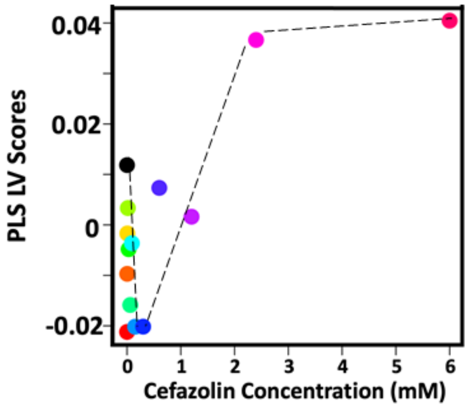

Figure S5: *Partial least squares regression analysis (PLS-R) latent variable LV2 scores corresponding to the loadings described in the main text (Fig.6), produced from Verification dataset. Each data point is the average of triplicate measurements, where black is serum only and colors denote addition of drug.*

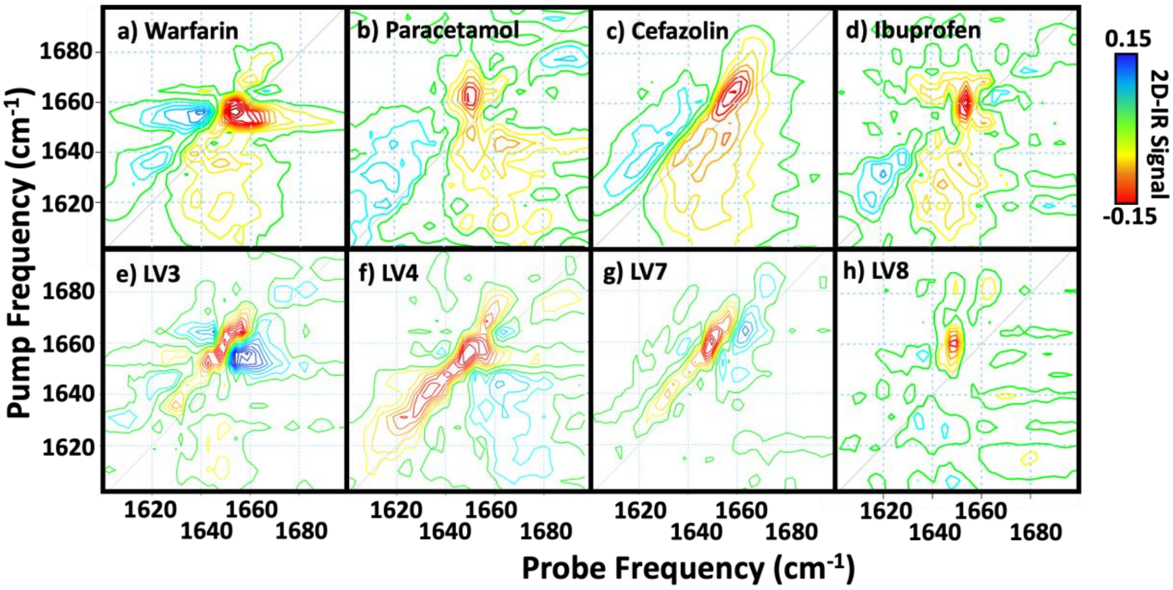

Figure S6: *2D-IR difference spectra obtained via subtraction of a neat serum spectrum (Fig.1) from the spectra in Fig.2(a-d): a) warfarin, b) paracetamol, c) cefazolin sodium and d) ibuprofen. Loadings for latent variables (LV) e) LV3, f) LV4, g) LV7, h) LV8, following PLS-DA model described in main text. It can be seen that the main features in each case are broadly consistent, though the PLS-DA model identifies sources of variance, some of which may be common to more than one drug molecule.*

References

Wold, S.; Sjostrom, M.; Eriksson, L.; PLS-Regression: A Basic Tool of Chemometrics. *Chemometrics and Intelligent Laboratory Systems*, **2001**, *58*, 109-130
